# Priming Gestures with Sounds

**DOI:** 10.1371/journal.pone.0141791

**Published:** 2015-11-06

**Authors:** Guillaume Lemaitre, Laurie M. Heller, Nicole Navolio, Nicolas Zúñiga-Peñaranda

**Affiliations:** Dietrich College of Humanities and Social Sciences/Department of Psychology/Auditory Laboratory, Carnegie Mellon University, Pittsburgh, Pennsylvania, United States of America; University of Tokyo, JAPAN

## Abstract

We report a series of experiments about a little-studied type of compatibility effect between a stimulus and a response: the priming of manual gestures via sounds associated with these gestures. The goal was to investigate the plasticity of the gesture-sound associations mediating this type of priming. Five experiments used a primed choice-reaction task. Participants were cued by a stimulus to perform response gestures that produced response sounds; those sounds were also used as primes before the response cues. We compared *arbitrary* associations between gestures and sounds (key lifts and pure tones) created during the experiment (i.e. no pre-existing knowledge) with *ecological* associations corresponding to the structure of the world (tapping gestures and sounds, scraping gestures and sounds) learned through the entire life of the participant (thus existing prior to the experiment). Two results were found. First, the priming effect exists for ecological as well as arbitrary associations between gestures and sounds. Second, the priming effect is greatly reduced for ecologically existing associations and is eliminated for arbitrary associations when the response gesture stops producing the associated sounds. These results provide evidence that auditory-motor priming is mainly created by rapid learning of the association between sounds and the gestures that produce them. Auditory-motor priming is therefore mediated by short-term associations between gestures and sounds that can be readily reconfigured regardless of prior knowledge.

## Introduction

Sounds provide essential feedback for many everyday events: for example, sounds tell listeners that water is boiling in the pot, that a chunk of vegetable is stuck in the blender, or that a bottle is about to overflow. Even in the absence of visual input, listeners can identify some properties of the events causing the sounds [1–3]. For instance, several of the object properties that are typically thought of as visual, such as an object’s shape, size, and material, can be conveyed through the auditory channel in constrained circumstances [4–7]. However, recent research suggests that the auditory channel is superior at conveying properties of actions. For instance, listeners are better at identifying that a sound event was made by certain actions, such as objects being rolled or scraped, than they are discerning certain properties of the objects themselves, such as materials [2, 8]. Related research also shows that learning how to perform a complex action is facilitated when the action provides dynamic auditory feedback [9]. This is consistent with a small body of literature showing that sounds produced by human actions (e.g. tool use) play a role in action execution, potentially cueing or informing gesture planning and execution (see for instance [10, 11] and ensuing paragraphs for a discussion).

The goal of the present study was to explore the nature of the associations between auditory perception and action by asking how hearing a sound produced by a manual gesture can prime executing that gesture. The study compared arbitrary gesture-sound associations created by the experiment and ecological associations resulting from life-long exposure. The auditory system could, in principle, connect either to object knowledge, motion execution, implicit motor planning, or any combination of these. Because very few behavioral studies have investigated these issues specifically for auditory perception, we will preface our study by presenting the framework provided by two neighboring sources: models of auditory neural processing, and behavioral interactions between stimuli and gestures in the context of the ideomotor theory.

One model of auditory neural processing characterizes the auditory system as having both a ventral and a dorsal stream, mirroring the dual stream model for vision (see [12, 13] for a review of this division). The auditory ventral stream (the “what pathway”) handles invariant object properties, phonetic access, and semantic processing via projections from primary auditory areas toward temporal and inferior frontal regions. In contrast, the auditory dorsal stream (the “how” and “where” pathway) handles language comprehension, perception of movement, multisensory integration, and the spatial properties of actions via projections toward temporal and parietal regions [14–18]. Parameters and details of motor plans are elaborated in the dorsal pathway, including parietal regions that process on-line motor codes for the interaction of limbs in relation to objects [19–21].

Sensory stimuli may interact with actions via either of these two pathways. For instance, Tucker and Ellis [22] have suggested that the visual compatibility effect (detailed below) may actually result from two types of representations generated by stimuli: (1) on-line details of the pragmatic aspects of action execution in the dorsal stream, and (2) stable knowledge in long-term memory about how to manipulate an object in the ventral stream. Similar types of interaction may occur in the auditory system as well. Results from neuroimaging studies clearly support the above possibilities [21]. Functional MRI studies compared neural activation associated with sounds made by human actions (typically, sounds of tools in use) to control stimuli (such as environmental sounds not made by human actions, animal vocalizations, etc. [23–28]). They have repeatedly found two regions: inferior parietal regions (i.e. dorsal stream) and the left ventral premotor cortex (i.e. ventral stream), forming the fronto-parietal network of sensory-motor processing.

Given the existing evidence for neural activation of motor regions by sensory stimuli, it seems logical that sensory stimuli may interact behaviorally with action planning and execution. Examples of such behavioral interaction come from the study of visually guided actions, stimulus-response compatibility effects (SR), and response-effect compatibility (RE).

Research on visually guided actions has shown that the affordances of a visual stimulus and the intentions of the participant can influence the participant’s actions [22, 29–34]. In a typical paradigm for instance, participants respond to a picture of an object by selecting a precision or power grip, depending on whether the object is natural or man-made [35]. Reaction times are faster when the size of the object is compatible with the grip than when it is incompatible. To our knowledge however, this paradigm has not been applied to auditory perception. In fact, there are few ecological situations in which a sound may afford or guide an action. For instance, a person may catch a ball rolling down a ramp just by listening to the sound of the ball [36], and visually impaired athletes can catch and hit baseballs if the ball emits beeps [37]. But for the most part, environmental sounds typically occur *as a consequence* of gesture (i.e. *after* the gesture) and are thus not used to adjust action parameters during gesture planning.

Nonetheless, it is possible that the contingency between a gesture and its acoustic consequence can create a strong association between gesture and sounds as illustrated by studies of the stimulus-response compatibility effect. In typical SR paradigm, a participant selects a *target response gesture* in response to a task-relevant property of a *stimulus* (i.e. choice-reaction task), whereas some other, task-irrelevant properties of the stimulus influence the gesture. A classic example is the Simon task, in which participants respond to a verbal cue (“left” or “right”) by pressing a left-hand or right-hand key [38]. The lateralization of the verbal cue, although task irrelevant, is then shown to prime the response: either speed up (when congruent with the lateralization of the response) or slow down the response (when incongruent). More generally, the SR effect is driven by dimensional overlap between stimulus and response, and has been demonstrated for auditory stimuli as well. For instance, Kunde et al. (2002) have shown that the duration of auditory stimuli can affect the priming of short and long key presses [39]. Even more relevant is the study by Hommel (1996) [40] who used tones to prime a choice-reaction task. In this case, each key produced either a low-pitched or a high-pitched tone, and an initial learning phase exposed participants to this association (i.e. associative learning). During the test phase, the tones were played *before* the stimulus. Results showed that the tones primed the responses. Our study used this latter paradigm because it uniquely separates the irrelevant priming stimulus (the tone) from the relevant movement instruction (which finger to press) rather than integrating the priming stimulus into a dimension of the movement cue.

Quite remarkably, RE paradigms have shown in addition that the prime need not be physically present before the response. They have shown that the *anticipated* consequence of a response also influences the selection and initiation of that response [41–43]. Participants respond faster when the consequences that *follow* the responses are congruent with the responses (e.g. quiet or loud tones following soft or forceful key presses versus the opposite).

Overall, the reviewed results support the *ideomotor* theory [44]. The central tenet of ideomotor theory is that motor plans specifying an action are associated in memory with the *consequences* of that action in a distributed and bidirectional relationship (consisting of any perceivable consequences of an action, proximal or distal, including proprioceptive, haptic, visual, or auditory consequences). In addition, ideomotor theory posits that activating any element of such a distributed representation may activate the whole representation: for instance, activating endogenously (i.e. RE compatibility) or exogenously (i.e. SR compatibility) the sensory consequences of an action may activate the motor plans producing that action and thus trigger or prime that action. The ideomotor theory is distinct from the sensorimotor approach, which treats actions as responses to stimuli, whereas the ideomotor approach tends to treat actions as following from internal causes such as goals. The *theory of event coding* combines these two approaches by treating reactions to stimuli as goal-directed actions [45]. It posits a common representational domain for stimuli (perceived events) and goals (intended events). Different features in this representation can be flexibly weighted depending upon the observer’s task, intentions, and perceptions [46].

A central tenet of the ideomotor theory is that once the association between an action and its consequences is acquired, people use it to control their actions by anticipating their sensory consequences. To be used in planning actions, the acquired associations must be very strong and presumably robust despite variations in experience over time. And yet, some plasticity must remain, because the sounds a person experiences when executing an action can change dramatically with age for instance (singing and speaking being a prime example). Another example comes directly from the studies of RE compatibility, whereby the putative long-term associations (e.g. a forceful key press triggering a loud tone) conflict with new associations created during the experiment (e.g. a forceful key press triggering a quiet tone) [43].

The plasticity of such associations has been little studied. As a matter of a fact, most of the experimental studies of the SR effect use *arbitrary* gesture-sound associations (participants have never encountered these associations before the experiment) that are rapidly created *during* the experiment. As such, the observed effects rely on very recent gesture-sound associations. In Hommel’s study for instance [40], the gesture-sound association does not commonly occur in the world: participants are exposed to the associations during the learning phase (associative learning), and the association is further reinforced during the test phase.

Other studies in the visual domain used the *ecological* consequences of an action [47–50]: In this case, the gesture-visual association corresponds to the structure of the world and thus participants have a stable knowledge of this association prior to the experiment (from repeated exposure throughout their entire life). For instance, Brass et al. (2000) used videos of fingers moving to prime finger gestures [47]. Similarly, gesture priming by sound may also draw upon prior experience and long-term representations of ecological associations (i.e. the natural consequences of an action). For example, we have previously shown that listeners can accurately and very quickly recognize the gestures (e.g. hitting or scraping a surface) that produce certain sounds [2]. It is therefore plausible that cognitive and perceptual representations of such sounds may be strongly associated with the general class of actions that produce them. The only related study to our knowledge that used *ecological sounds* was reported by Castiello et al. [11] (using a go/no-go task). Participants reached for a sphere in response to a go signal. The sphere maintained the same dimension and the same weight, but was covered with different materials on different trials (foil, wool, etc.). A priming sound (i.e. the contact sound made by fingers grasping the sphere) was played before the response or while participants were performing the gesture). In both cases, the duration (*movement time*) of the reaching gesture was reduced when the prime sound was congruent with the material of the sphere. However, it is unclear if the change of movement times resulted from priming or from a mismatch between simultaneous visual and auditory information.

The goal of the present study was to assess the plasticity of the gesture-sound associations by comparing priming gestures with sounds that were either arbitrarily or ecologically associated with these gestures in a choice reaction-time task. By the previous line of reasoning, ecological associations should yield more robust priming effects than newly created associations. This assumes that the priming effect draws upon stable long term associations that take time to establish and to update. In addition, ecological associations should persist more durably than newly created associations once the association is no longer reinforced. An alternative possibility is that the priming effect is mediated by short-term, plastic gesture-sound associations, in which case ecological and arbitrary associations should yield the same priming effects, and priming should disappear quickly once the association is no longer reinforced for both types of associations.

**Fig 1:**
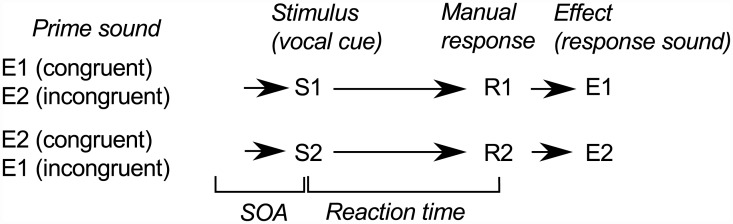
The structure of a trial, common to all experiments. Participants responded to vocal cue S1 or S2 by executing a response gesture R1 or R2. R1 and R2 produced response sounds E1 and E2, respectively, which were also used to prime the gestures. R1 and R2 are mapped to left-hand and right-hand key lifts in Experiment One and Experiment Two and mapped to tapping and scraping gestures in Experiments Three and Four. E1 and E2 are low-pitched and high-pitched tones in Experiment One whereas they are tapping and scraping sounds in Experiments Two and Three. See Table 1 for a design summary.

All experiments followed the same stimulus-response paradigm summarized in Fig 1: participants responded to a vocal cue (S1 or S2) by executing a response gesture (R1 or R2) that produced a response sound (E1 or E2). Response sounds were used as primes during the test phase. Experiment One used a paradigm similar to Hommel’s with key lifts and tones. Experiment Two replicated it with the tapping and scraping sounds of Experiment Three associated with key lifts. Experiment Three used a custom interface in which participants performed tapping and scraping gestures. The sounds directly produced by these actions were recorded prior to experimentation, and these recordings were used as primes. Experiment Four replicated Experiment Three with no learning phase, and the interface was muffled so that the two response gestures produced no sounds. Finally, Experiment Five replicated Experiment One (key lifts and tones), but stopped the response sounds halfway through the experiment to observe if the priming effect can persist even when the gesture-sound association is no longer reinforced. Each experiment used a different set of participants to ensure that learning the gesture-sound association did not carry over across experiments. Data are available at the following URL: https://zenodo.org/record/20734.

## Experiment One: priming key lifts with tones

Experiment One was a conceptual replication of a study by Hommel [40]. Participants responded to a verbal cue (“left” or “right”) by lifting a left-handed or right-handed key (we chose to use key lifts instead of key presses to avoid activating any preexisting associations between key-presses and impact-like sounds). Each key lift was accompanied with an experimenter-generated response tone. Prime and response sounds consisted of a low-pitched and a high-pitched tone. Such sounds are commonly associated with key presses in various devices (ATM, cell phones, etc.), but there is no ecological association of certain pitches to certain keys of a keyboard. The only potential systematic association we are aware of is that of the keyboard of musical instruments where pitch increases from left to right [51]. To exclude such a bias, the assignment of gesture and tones was counterbalanced. Participants were trained to associate the tones and the key lifts in a preliminary learning phase.

### Method

All participants provided written consent to participate in this study (as well as the participants of subsequent experiments). This and the subsequent studies were approved by Carnegie Mellon University Institutional Review Board (HS15-408).

#### Participants

The 37 right-handed participants were split into two groups of participants to counterbalance the assignment of a key to a response sound. Nineteen English-speaking participants (7 female, 12 male) between the ages of 18 and 21 (median 19 years old) formed Group One. Eighteen English-speaking participants (9 female, 9 male) between the ages of 18 and 22 (median 19 years old) formed Group Two. Participants in all experiments had self-reported normal hearing and right handedness.

#### Stimuli and Apparatus

This experiment used an Apple USB keyboard (Model No: A1243). The digital files were converted to analog signals by an Audiofire 4 audio interface. The directional cues and the primes were presented over Sennheiser HD600 headphones.

#### Stimuli

Both the vocal directions “Right” and “Left” were recorded from a native American English speaker via an Audio-Technica AT3525 30 Series microphone in IAC double-walled sound-attenuating booth. We equalized the durations of the two cue words. The recorded primes were presented at a level similar to that of the response sounds (about 80 dB).

The low-pitched tone was a 534-Hz sinusoid and the high-pitched sound was a 1730-Hz sinusoid. Both sinusoids were multiplied by an Attack-Decay-Sustain-Release envelope (ADSR), with an attack time of 5 ms, decay time of 10 ms, sustain duration of 50 ms, and release time of 5 ms (total duration = 70 ms). During the sustain portion, the tones remained at 50% of the maximum amplitude. Sounds were played at a 44100-Hz sample rate with 16-bit resolution.

The high-pitched tone was associated with the left key and the low-pitched tone to the right key for the first group. This mapping was reversed for the second group. For the sake of simplicity, the sound associated with the left key in each group (high-pitched tone in Group One, low-pitched tone in Group Two) will be reported as the “left sound” and the sound associated with the right key (low-pitched tone in Group One, high-pitched tone in Group Two) will be reported as the “right sound”.

### Procedure

The structure of a test trial is represented in Fig 1. Each trial started with the participants placing their hands on the “home” position. A prime sound or a silent interval started 400 ms later. This prime was followed by a vocal cue indicating which response to execute (indicated as “S1” or “S2” in Fig 1). Participants began all of the trials in “home position”, which was simply using their left and right index fingers to simultaneously hold down the left key (“control” key on the bottom left of keyboard) and the right key (“enter” key on the bottom right of the keyboard). They responded to the directional cues by lifting their finger from the left key (“R1”) or the right key (“R2”). A low-pitched or a high-pitched response tone (“E1” or “E2”, depending on the group) was immediately played when they lifted each key. They were instructed to respond as rapidly as possible and could respond as soon as the directional cue began.

The stimulus onset asynchrony between the prime sound and the directional cue was set at 135 ms based on preliminary experiments. Reaction times were measured from the onset of the direction cue to the time when participants moved their hand away from the home position.

The prime sounds provided no information as to which gesture would be required. Half of the trials cued the participants to lift the left key and half of the trials cued the participants to lift the right key. One third of the trials used a prime congruent with the cued response gesture. One third used an incongruent prime. One third used a silent prime (baseline) that had a silent gap of the same duration as the other primes. There were therefore six types of trials (two response gestures times three primes). A total of 324 trials were presented to each participant in 18 blocks of 18 trials each. After each trial a recorded vocal message indicated if the answer was correct. After each block, visual feedback was displayed on the computer monitor indicating the percent of correct answers and average reaction time along with vocal encouragement to speed up. An equal number of each of the six trial types was presented, in random order, within every three-block sequence (54 trials). This structure ensured an equal distribution of trial types throughout the experimental session.

Prior to the main session, the experimenter demonstrated the procedure to the participant for 24 trials. Next, the participants familiarized themselves with the experiment in a preliminary training session. They conducted four blocks of 18 trials in the presence of the experimenter. During this training session the participants interacted with the experimenter to clarify the procedure. The experimenter ensured that the participants executed the correct gestures, responded correctly to the cues, and were responding as quickly as they could. Next, the participants conducted practice trials on their own. Prime sounds were always present during the demonstration and training phases.

After these training trials, participants performed the learning phase (324 trials). The learning phase was identical to the main test phase, without prime sounds. Finally, they performed the test phase (324 trials with prime sounds).

### Results

We measured both response accuracy (reflecting the percent of trials in which the correct response gesture was made) and reaction time (reflecting the time to initiate the response gesture after the onset of the direction cue). Only participants who reached a minimum 85% accuracy across trials were considered in the analyses. Accuracy and relative reaction times (see below) were submitted to a three-way ANOVA with the response gesture and the congruency between prime and response sounds as the within-participant factors and the groups as the between-participant factors, similar to the analyses reported by [40]. Analyses of accuracy considered the congruent, incongruent, and no-prime conditions. Analyses of reaction times considered only the congruent and incongruent conditions, because the no-prime trials were used to compute the relative reaction times (see below). All analyses were subjected to a Geisser-Greenhouse correction due to a possible violation of sphericity when necessary; p-values are reported after correction. Planned contrasts used Pillai’s test. Post-hoc t-tests are also reported after Bonferonni correction when necessary. The design and effect sizes are summarized in Table 1 along with those of next experiments.

**Table 1:** Outline of the experimental design for the five experiments. See Fig 1 for the structure of a trial. *: p<.05. **: p<.01. n.s.: non-significant.

Experiment	S1/S2	R1/R2	E1/E2	Learning	Effect size
One	“Left”/“right”	Left/right key lifts	Low/high tones	Yes	23.0 ms**/ 4.4%**
Two	“Left”/“right”	Left/right key lifts	Tap/Scrape	Yes	17.9 ms**/ 2.0%**
Three	“Left”/“right”	Tapping/scraping	Tap/Scrape	No	15.9 ms**/ 1.7%**
Four	“Left”/“right”	Tapping/scraping	None	No	5.5 ms*/1.0%**
Five	“Left”/“right”	Left/right key lifts	1: L/h tones	Yes	20.9 ms**/3.2%
			2: None		2.4 ms (n.s.)/0.6%

#### Reaction times

Reaction times (RTs) were preprocessed in two steps. The first step removed trials that were incorrect. The second step of preprocessing removed outlier RTs. The cutoff to exclude outlier was established in each experiment so that less than 0.5% of trials were excluded [33]. The cutoff was set to 840 ms in Experiment One (0.47% of trials). Note that a different cutoff was used for every analysis, to ensure the exclusion of no more than 0.5% of the trials. After preprocessing, each participant’s mean RT for each type of trial was calculated.

We addressed the fact that the RTs would not be equivalent for the two gestures by including the “no prime” condition as a separate baseline measure for each gesture. Thus, we calculated *relative* RTs by subtracting out the RTs for the baseline (silent prime) for each gesture and for each participant. However, this measure is systematically negative since participants responded slower when there was no prime. We therefore added to this number the mean RT for the two primes, averaged across all participants and conditions. The resulting relative RT is the average RT from trials with a prime *for a given gesture and a given prime* minus the RT for the baseline *for the same gesture* plus the average RT for any prime. The goal of this step was to produce positive numbers with the same average as the unprocessed RTs, rendering them easier to interpret than negative relative measures. Note that adding a constant to the dependent variable does not change the results of an ANOVA (see [52], p. 453–455).

**Fig 2:**
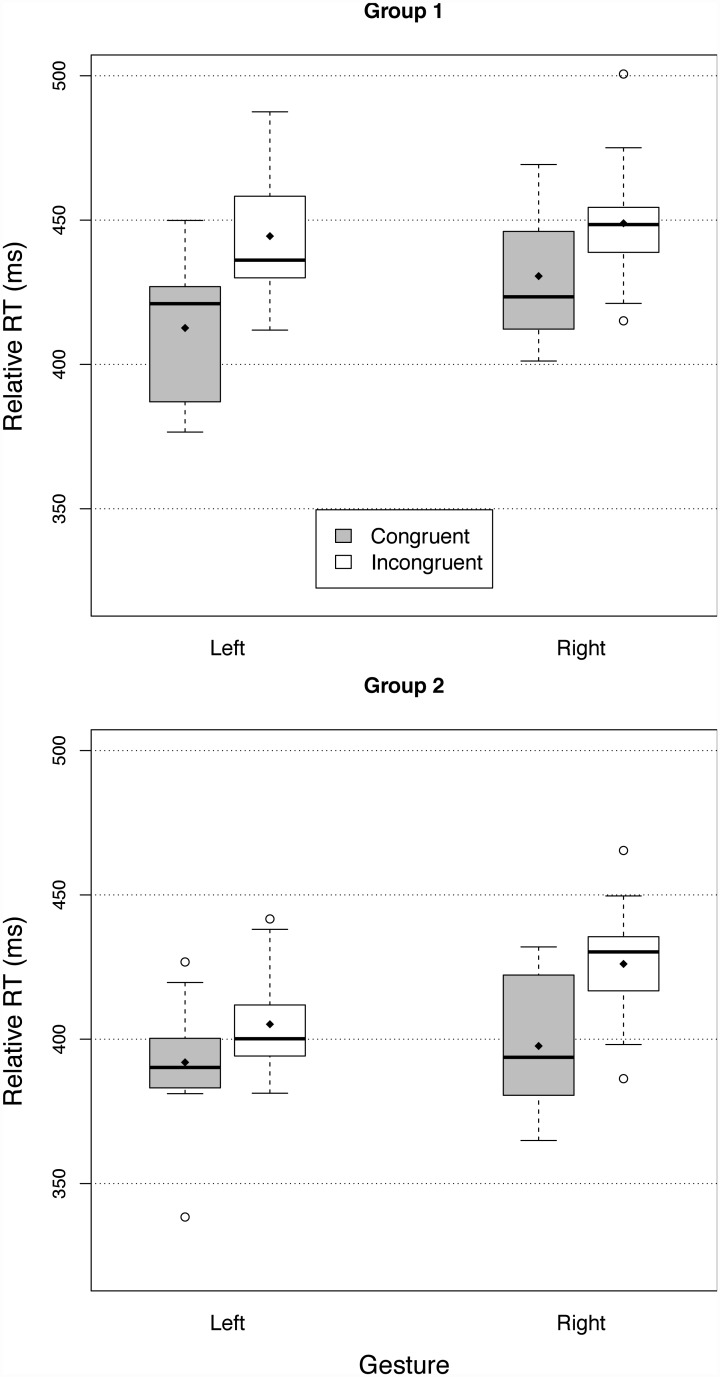
Relative reaction times in Experiment One (lifting a finger to initiate a high-pitched or a low-pitched tone)—with Group One (left hand high tone, top panel) and Group Two (left hand low tone, lower panel). The solid box represents the interquartile (IQ) range of the distributions of RTs. The dashed lines represent the interquartile range plus or minus 1.5 IQ. Circles represent data points outside this range (i.e. outliers). The horizontal lines represent the median and the losanges the means of the distributions.

Reaction times are represented in Fig 2. The main effect of congruency was significant (F(1,35) = 38.75, p<.0001, generalized *η* ^2^ = 0.25 [53]), and did not interact with the response gestures (F(1,35) = 0.05, p = 0.83), nor with the groups (F(1,35) = 0.33, p = 0.58). Across groups and gestures, relative RTs were 23.0 ms smaller for congruent primes than incongruent primes. Overall, relative RTs were 28.1 ms shorter in Group 2 than in Group One, (F(1,35) = 35.90, p = <.0001, *η* ^2^ = 0.346). There was a significant difference between the two response gestures (F(1,35) = 33.70, p<.01, *η* ^2^ = 0.087) but the effect of the gestures did not interact with the groups (F(1,35) = 0.24, p = 0.63). Across groups, participants responded 12.2 ms faster with the left than the right gesture. The three-way interaction between congruence, gesture, and group was significant (F(1,35) = 13.5, p<.001, *η* ^2^ = 0.032), though much smaller in size than the other effects. This interaction was driven by a difference of priming size between the two gestures in each group. In Group One, the difference between congruent and incongruent primes was larger for the left gesture (31.8 ms) than for the right gesture (18.2 ms). In contrast, in Group Two, priming size was smaller for left gesture (13.2 ms) than for right gesture (28.4 ms). In other words, priming was always smaller for the gesture that was associated with the low-pitched response tone. Although the tones were played at the same level in dB, they had different frequencies, and thus there probably were slight differences in loudness between the two priming sounds [54], which may have affected the size of the the priming effect.

#### Accuracy

Overall, accuracy was high, with an average of 94.6%. The main effect of congruency was significant (F(2,70) = 42.01 p<.0001, generalized *η* ^2^ = 0.283) and did not interact with the groups (F(2,70) = 1.38, p = .26). Across groups and response gestures, participants made fewer errors with the congruent prime (95.7% correct) than the incongruent prime (91.3%, F(1,35) = 37.71, p<.0001). They also made fewer errors when there was no prime (96.9% correct, F(1,35) = 49.44, p<.0001) than when there was a prime (93.5% correct).

There was a small but significant difference in accuracy between the groups (F(1,35) = 6.20, p<.05, *η* ^2^ = 0.054). Participants in Group One made slightly more errors (93.7% correct) than in Group Two (95.6% correct). The main effect of the cued gestures was also significant (F(1,35) = 10.46, p<.01, *η* ^2^ = 0.036) and did not interact with the groups (F(1,35) = 0.24, p = 0.63). Participants made more errors with the right key (93.9%) than the left key (95.4%).

The interaction between congruency and response gesture was significant (F(2,70) = 4.05, p<.05, *η* ^2^ = 0.025), and the three-way interaction between congruency, response gesture, and group was not significant (F(2,70) = 0.95, p = .39). This effect was much smaller than the main effect of interest (congruency *η* ^2^ = 0.283).

### Discussion

Experiment One confirmed Hommel’s findings [40]: An arbitrary sound contingently associated with a gesture influences the time to select that gesture when used as a prime. Because there is no reason to believe that a previously learned association existed prior to the experiment, the association must have been created by the learning phase at the beginning of the experiment and reinforced by the experiment itself. (The possibility of a musical association between the left-hand side of the keyboard and low pitches and vice versa was addressed by counterbalancing associations in the two groups.) Here, priming was observed both for accuracy and reaction times.

In addition to the primary comparisons, it is worth noting that there were a few asymmetries found in secondary comparisons. Participants made fewer errors and responded faster when they responded with the left hand. Counterbalancing ruled out any effect of the particular response sounds. It cannot be attributed to the latency of the keyboard since only relative RTs were considered. Across counterbalanced groups, the size of the priming effect was also larger for the key associated with the low-pitched sound (left key in Group One, right key in Group Two). Such asymmetry is difficult to interpret and orthogonal to the main interest of this study.

The mere presence of an auditory priming sound also sped up the participants’ responses on average, suggesting that the prime sounds alerted participants that the directional cue was about to be played. This alerting function was subtracted out when two primed conditions were compared and therefore did not play a role in any of our critical comparisons. Because we used a silent prime rather than a “neutral” prime, it was not possible to separate facilitation from inhibition in our results. *We chose not to try to use a “neutral” cue because any sound could potentially be more associated with one gesture than another.* Therefore, all experiments used the silent baseline. All of our causal claims focus on the relative advantage of prime-gesture congruence.

## Experiment Two: priming key lifts with tap and scrape sounds

Experiment Two followed the same general procedure as Experiment One, except for one major difference: The response and the prime sounds were the tapping and scraping sounds that will be used in Experiment Three instead of tones (described in detail there). Similarly to Experiment Three, Experiment Two did not counterbalance the mapping between response gestures and response sounds. The goal of this experiment was thus to facilitate comparison with Experiment Three by using the same design and same response sounds.

### Method

#### Stimuli, Apparatus, and Procedure

This experiment used an Apple USB keyboard (Model No: A1243). The procedure, directional cues, primes, and other pieces of equipment were similar to that of Experiment One. Prime and response sounds were the tapping and scraping sounds used in Experiment Three.

#### Participants

Thirty English-speaking participants (18 female, 12 male) between the ages of 18 and 21 (median 19 years old) took part in the experiment. All were right-handed.

### Results

The same analyses as in Experiment Two were carried out in Experiment One. The design and effect sizes are summarized in Table 1.

#### Reaction times

**Fig 3:**
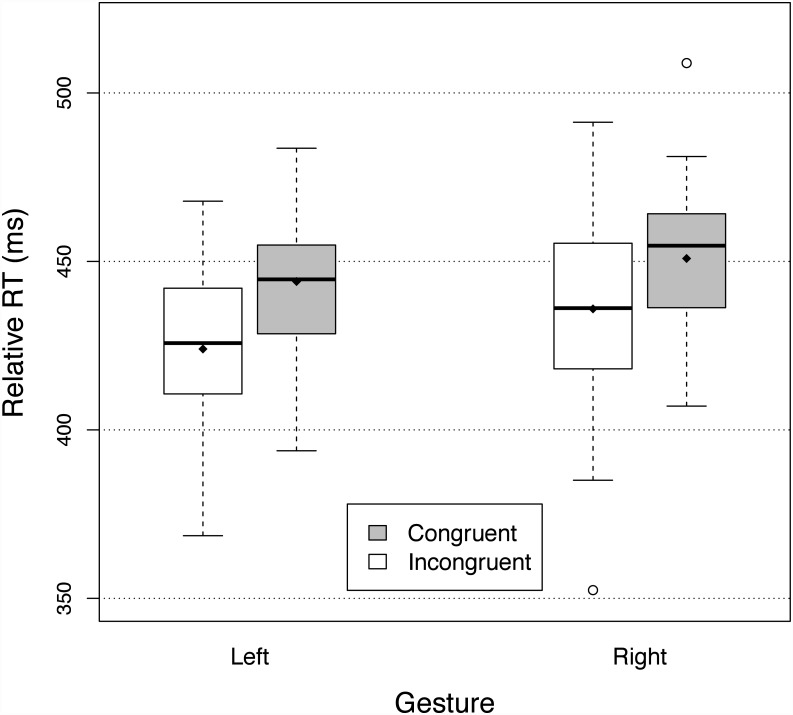
Relative reaction times in Experiment Two (lifting a finger to initiate a scraping or tapping sound). See legend of Fig 2 for details.

Reaction times were preprocessed as in Experiment One, with a 870-ms cutoff to exclude outliers (0.49% of trials). As in Experiment One, RTs were longer for the baseline than the primed conditions and we used the relative RTs as a dependent variable. A two-way full-factorial ANOVA showed a significant effect of congruency and showed that relative RTs were 17.9 ms smaller for the congruent primes than the incongruent primes (F(1,29) = 56.31, p<.0001, *η* ^2^ = 0.105). There was no significant interaction between the primes and the gestures (F(1,29) = 1.04), p = 0.32), indicating that the size of the priming effect did not depend on the gestures. Relative RTs were significantly different between the two response gestures (F(1,29) = 8.27, p<.01, *η* ^2^ = 0.034), with relative RT being 7.0 ms larger for the right key than for the left key. These results are reported in Fig 3.

#### Accuracy

Overall, accuracy was high, with an average of 94.1%. The main effect of the cued response gesture was significant (F(1,29) =17.49, p<.001, generalized *η* ^2^ = 0.068) and showed that participants were more accurate with the left (95.2%) than the right key (93.0%). The main effect of congruency was also significant (F(2,58) = 7.49, p<.01, *η* ^2^ = 0.079). Planned contrasts showed that participants were more accurate with the congruent primes (94.5% correct) than with the incongruent primes (92.5% correct, F(1,29) = 8.024, p<.01), and that they were more accurate with no prime (95.3%) than with the primes (93.5% correct on average, F(1,29) = 7.077, p<.05). The interaction between the cued gesture and the prime was not significant (F(2,58)= 1.16, p = .32).

### Discussion

The results of Experiment Two replicated Experiment One with another set of response sounds: the sound associated with a gesture can serve to prime the production of that gesture, even when the sound-action association is arbitrary and newly learned during the course of the experiment. The size of the priming effect did not depend on the hand that was used to respond. But in all primed conditions, participants responded slightly more rapidly and more accurately with the left key than with the right key. Such an asymmetry is difficult to interpret. It cannot be attributed to the latency of the sensors or a difference between the directional cues since *relative* RTs are calculated for each case. Nor can it be easily attributed to the differences between the primes since this effect is observed when averaging across RTs for both primes. Furthermore, this small effect of hand side has no interaction with the effect of the prime congruency.

Experiments One and Two demonstrated priming created by new associations between prime sounds and response gestures that were not ecologically related. Experiment Three addressed the question of whether ecologically associated sounds and gestures show any unique priming effects.

## Experiment Three: priming tapping and scraping gestures with taps and scrapes

Experiment Three used common gestures and priming sounds that are ecologically produced by these gestures (tapping and scraping, with the corresponding ecological sounds as the primes). We hypothesized that the association between sounds and gestures should be strongly integrated by participants if there is priming that relies on long-term gesture-sound associations. Therefore, the motor priming should be readily observed even in the absence of a learning phase.

### Method

#### Participants

Thirty English-speaking participants (22 female, 8 male) between the ages of 19 and 59 (median 22 years old) took part in the experiment. All were right-handed.

#### Response interface and apparatus

This experiment used a custom response device (the “wooden interface”). The device consisted of a raised flat surface upon which two handles rested, with LED sensors, a guiderail, and a microchip (National Instruments USB Daq). Two sensors were embedded into the right side of the device’s face. The first sensor was at the “home” position for the right handle. The second was a few inches to the right of the first, along the path of the right handle. A guiderail piece was placed parallel to the path between the two sensors, against the back of the surface. This guided the right handle along the correct scraping path. Attached to the back of the left side of the device was a vertical piece of wood into which was embedded one more sensor, positioned about half an inch above the interface surface. This sensor detected when the left handle was in the home position.

The interface was made almost entirely out of Birch plywood and Oak dowels. The surface where participants scraped the right handle was covered with an aluminum board that had been grated so as to produce a consistent scraping sound. The sensors were comprised of infrared-emitting and infrared-sensing LEDs. These functioned by emitting infrared light and then detecting any of the light reflected off of a handle.

The other pieces of equipment were similar to those used in the previous experiments.

#### Stimuli

The primes were recordings of the tap and scrape sounds produced by the two response gestures. The sounds were recorded with a 96-kHz sampling rate and 24-bit resolution via a Tucker Davis Technologies MA3 microphone pre-amplifier, a Sound Devices USBPre soundboard, and an Earthworks QTC 30 microphone in an IAC double-walled sound-attenuated booth.

The response sounds that were ecologically produced as a consequence of making the response gestures were clearly audible through the open metal mesh headphones.

#### Procedure

Participants responded to the directional cue “left” by using the left hand to rapidly lift and lower the left handle, producing a single tap. Participants responded to the directional cue “right” by using the right hand to rapidly slide the right handle towards a target location further to the right, producing a scrape. Each response gesture produced a response sound that was an ecological consequence of the event. There was only one group of participants in this experiment (all participants tapped with their left hand) because it was not possible to reverse the response interface.

The stimulus onset asynchrony between the prime sound and the directional cue was set at 215 ms based on preliminary experiments (it was 135 ms in Experiment One and Experiment Two). This ensured that the perceived stimulus onset asynchrony was the same for the different types of primes. For instance, the scrape and tap sounds have gradual and sharp onset ramps, respectively, so matching the onsets of their waveforms would not necessarily result in equal perceived onset times. (cf. [55, 56] found that perceptual onsets determined isochrony rather than acoustic onsets.) Reaction times were measured from the onset of the direction cue to the time when participants moved their hand away from the home position.

A total of 396 trials were presented to each participant in 18 blocks of 22 trials each. Participants went through the same demonstration and training sessions with and without the experimenter as in Experiment Two, but there was no learning phase. The training sessions included the primes in this experiment, because we suspected that the training sessions could somehow teach the participants the gesture-sound associations. Including primes in the training sessions allowed us to measure the presence of a priming effect evolving during training.

### Results

The design and effect sizes of Experiment Three are summarized in Table 1. Data were excluded from one participant who did not complete the last block of the experiment.

#### Reaction times

Reaction times (RTs) were preprocessed with the same procedure previously described, with an 1.35-ms cutoff.

Due to differences in sensor placement as well as inherent speed differences between the two gestures, the two gestures had different latencies: participants were about 18 ms faster to initiate the tapping motion than the scraping motion. As in the previous experiments, subtracting out the RTs for the baseline (silent prime) for each gesture removed these differences from the analyses.

**Fig 4:**
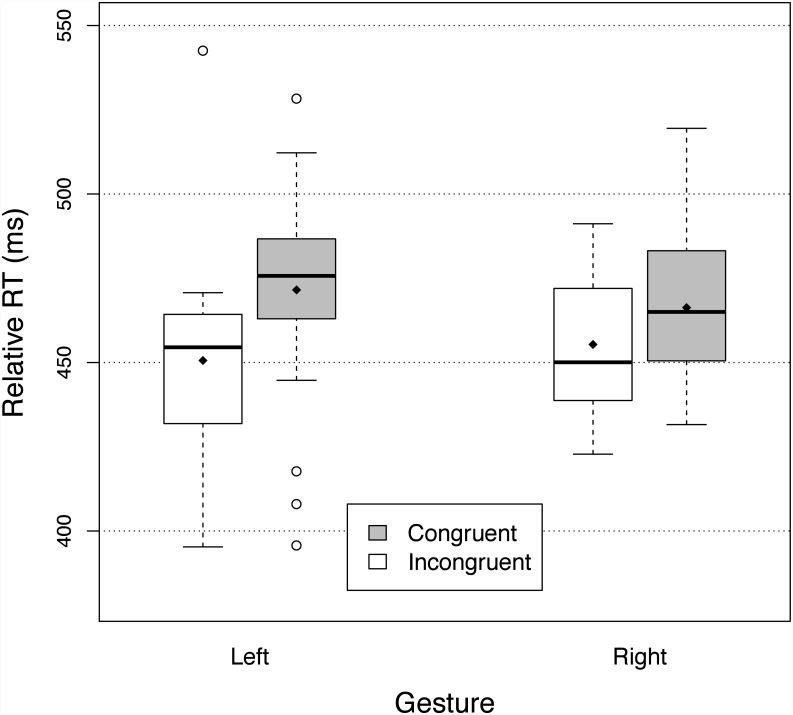
Relative reaction times in Experiment Three (tapping and scraping a wooden interface). See legend of Fig 2 for details.

Fig 4 shows the relative reaction times for the two gestures and the congruent and incongruent primes. There was a significant effect of congruency (F(1,28) = 32.09, p<.0001, *η* ^2^ = 0.099), with relative RTs being 15.9 ms faster for the congruent primes than incongruent primes. Interaction between the primes and the type of gestures was not significant (F(1,28) = 3.34, p = .08), indicating that priming was equivalent for both gestures. The relative RTs were not significantly different between the two response gestures (F(1,28) = 0.0033, p = .95).

**Fig 5:**
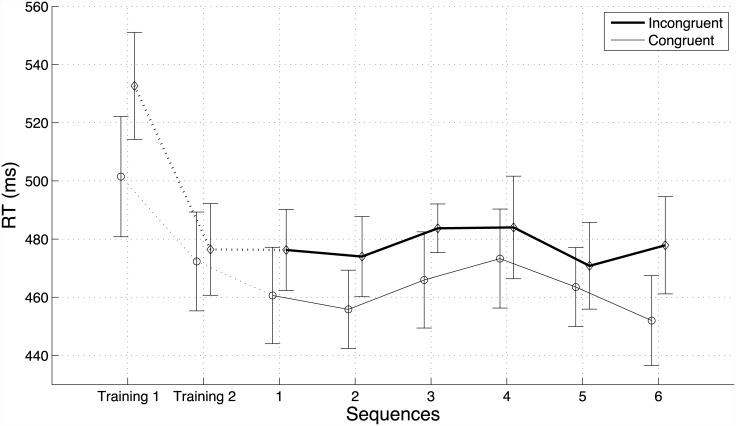
Relative reaction times in Experiment Three (tapping and scraping a wooden interface) averaged across the two response gestures, for the six sequences of three blocks, and for the training session. Error bars indicate the 95% confidence interval. Response sounds were always present.

Fig 5 represents the relative RTs averaged across the two response gestures, for the six sequences of three blocks (66 trials) used for randomizing the test trials and for the two training session (first training = 72 trials with the experimenter, training 2 = 72 trials without experimenter; data from two participants were lost and were excluded from the analysis of the training session). Reaction times were longer overall during the first training session (i.e. participants were still practicing the task). During the first training session, RTs were 31.2 ms shorter for the congruent than the incongruent trials (t(54) = 2.21, p<.05), and they were 4.1 ms shorter during the second training (t(54) = 0.35, p = 0.36). The size of the priming effect (RTs for incongruent trials minus congruent trials) was not significantly different between first and second training (32.2 ms vs 4.1ms t(54) = 1.83, p = 0.96), nor between the first training and the first chunk of the test session (15.6 ms, t(55) = 1.00, p = .16), nor between the first and the last chunk of the test session (25.9 ms, t(56) = 0.93, p = .82).

#### Accuracy

Overall, accuracy of response gesture was high, with an average of 96.4%. Accuracy data were submitted to a two-way full-factorial analysis of variance (ANOVA) with congruency condition (congruent, incongruent, no prime) and the two response gestures as repeated measures factors.

The main effect of congruency was significant (F(2,56) = 14.99, p<.0001, generalized *η* ^2^ = 0.078). Planned contrasts showed that participants performed significantly better for the congruent than the incongruent primes (96.6% vs. 95.0%, F(1,28) = 10.50, p<.01), and better for the baseline than for the two primes (97.6% vs. 95.8%, F(1,28) = 20.67, p<.0001). The interaction between cued gesture and congruency was not significant(F(2,56) = 2.39, p = 0.10). The main effect of the cued response gesture was not significant (F(1,28) = 1.03, p = .32).

### Discussion

Experiment Three showed that ecologically related sounds and response gestures can produce priming: the association between the sound and the gesture resulted from a common ecological physical phenomenon that participants have presumably encountered throughout their lives. In addition, Experiment Three did not use any learning phase. Analysis of the training session showed that the priming effect was actually already established during the first trials of the experiment and did not significantly evolve throughout the experiment (note that the limited number of trials in training sessions and randomization sequences decreased the power of our statistical tests.). As such, one hypothesis was that the priming effect could be thought of as resulting from participants’ previous experience with natural objects. But the priming effect may also have resulted from an association between sounds and gestures that was created *during* the training portion of the experiment and reinforced during the experiment, since every time participants performed the gestures, they heard the resulting sound. To test this idea, Experiment Four removed the response sounds. If the priming is mediated by long-term stable representations, removing the response sounds should not degrade the priming effect. Conversely, priming should not be observed if it relies only on short-term associations created during the experiment.

## Experiment Four: muffled scrape and tap sounds

Experiment Four followed the same general procedure as Experiment Three, except for one major difference: The handles and parts of the interface were “padded” with sound-absorbing foam to muffle the sounds that the participants were creating by performing the gestures. The directional cues and primes were the exact same as in Experiment Three. This allowed us to test whether hearing the response sounds was necessary in order to bring to mind the pre-existing associations between a gesture and its resulting sound.

### Method

#### Stimuli, Apparatus, and Procedure

This experiment used the interface described in Experiment Three, with sound-absorbing foam glued to the handles and parts of the interface to muffle the sounds that participants were producing when completing their gestures. The directional cues (“left” and “right”) and the primes (tap and scrape sounds) were the exact same as in Experiment Three. The procedure was identical to that of Experiment Three.

#### Participants

Thirty-one English-speaking participants (20 female, 11 male) between the ages of 18 and 57 (median 22 years old) took part in the experiment. All were right-handed.

### Results

The design and effect sizes of Experiment Four are summarized in Table 1.

#### Reaction times

**Fig 6:**
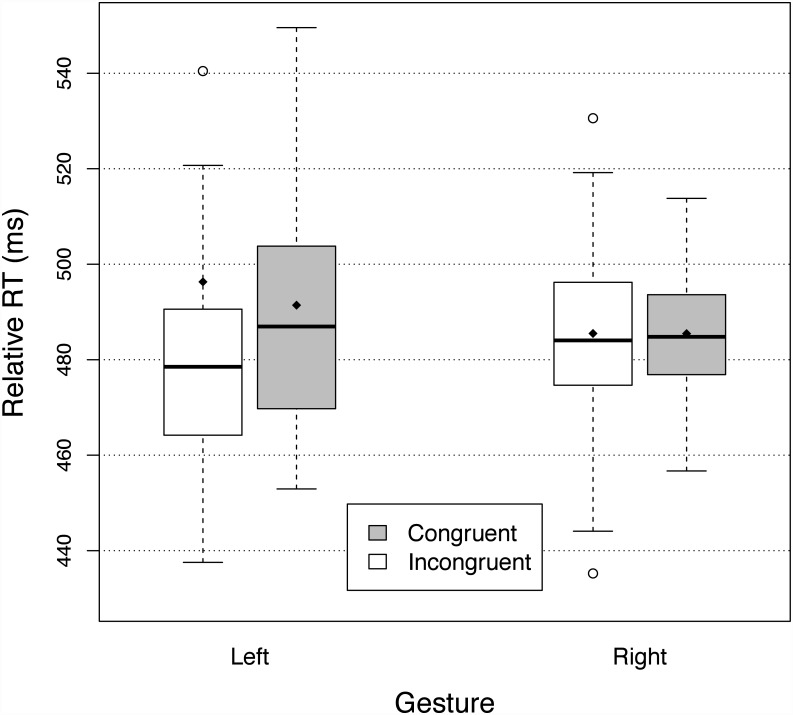
Relative reaction times in Experiment Four (tapping and scraping a muffled wooden interface). Error bars indicate the 95% confidence interval. See legend of Fig 2 for details.

Fig 6 represents the relative reaction times (RTs) for Experiment Four. RTs were preprocessed the same way as in the other experiments (cutoff 1230 ms). Analysis showed that relative RTs were significantly different between incongruent and congruent primes F(1,30) = 5.51, p = <.05, *η* ^2^ = 0.019 (the difference of relative RTs between incongruent and congruent primes is 5.5 ms), nor between the two response gestures (F(1,30) = 0.31, p = .58). There was no significant interaction between the primes and the type of gestures: F(1,30) = 2.31, p = .14).

### Accuracy

Overall, accuracy was high, with an average of 96.7%. The main effect of congruency was significant (F(2,60) = 18.52, p<.0001, *η* ^2^ = 0.122). The main effect of the cued response gesture was not significant (F(1,30) = 1.20, p = .28). The interaction between the cued gesture and the prime was also not significant (F(2,60) = 1.08, p = .35).

Planned contrasts showed that accuracy was higher for congruent (96.5% correct) than incongruent primes (95.5% correct, F(1,30) = 8.84, p<.01), and that accuracy was significantly higher for the silent baseline condition (98.0%) than for the prime conditions (95.6%, F(1,30) = 23.45, p<.0001).

### Discussion

In Experiment Four, priming was observed for accuracy scores and reaction times, although its size was greatly reduced (by two thirds). Priming was almost eliminated when the response sounds of that gesture were not heard when performing that gesture. These results suggest that the long-term associations between sounds and gestures, though they exist and resulted in a small priming effect, were playing only a small part in the priming effect observed in Experiment Three. Instead, it appears that short-term associations created during training and reinforced *during* the experiment had the greatest role. The consequence of this interpretation is that priming mediated by recent arbitrary gesture-sound associations should disappear very quickly when the association is no longer reinforced by the experiment. To test this idea, Experiment Five replicated Experiment One, but added a set of 18 blocks of trials in which the response sounds were removed.

## Experiment Five: stopping response sounds halfway through

Experiment Five was similar to Experiment One except that it appended 18 blocks of trials in which response sounds were removed.

### Method

#### Stimuli, Apparatus

This experiment used the stimuli and apparatus described in Experiment One.

### Procedure

Experiment Five used a procedure similar to Experiment One with two exceptions. First there was only one set of 72 training trials (with the experimenter). Second, another set of 324 test trials was added after the first set of 324 test trials. In this last set, there was no response sound when the participants lifted the keys (response sounds were replaced with a silence of equivalent duration).

#### Participants

Thirty-four English-speaking participants (14 female, 20 male) between the ages of 20 and 38 (median 23 years old) took part in the experiment. All were right-handed. Participants were divided in two groups (17 in Group 1, 17 in Group 2). Each group received a different key-tone mapping.

### Results

The design and effect sizes of Experiment Five are summarized in Table 1.

#### Reaction times

**Fig 7:**
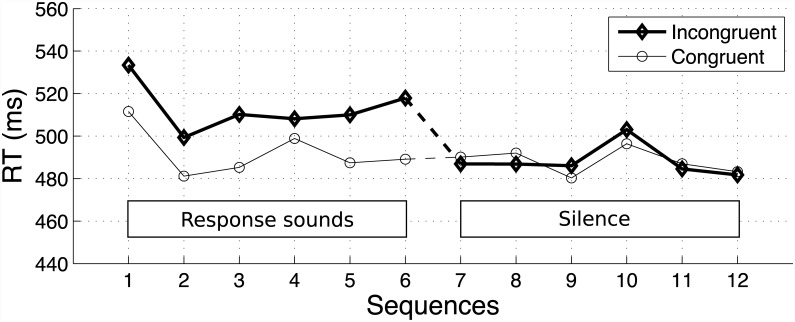
Relative reaction times in Experiment Five (key lifts and tones). Response sounds are played during sequences 1 to 6, and removed during sequences 7 to 12. Confidence intervals have been omitted for the sake of clarity.

Fig 7 represents the relative reaction times (RTs) for Experiment Five. RTs were preprocessed the same way as in the other experiments (cutoff 1350 ms). They were submitted to a four-way ANOVA, with the groups as a between-participant factor, and the congruency, the response gestures, and the randomization sequences (sequences 1 to 6—first half—with response sounds, sequences 6 to 12—second half—without response sounds) as within-participants factors. The main effect of congruency was significant (F(1,32) = 17.09, p<.001, generalized *η* ^2^ = 0.01) and did interact with the sequences (F(11,352) = 2.48, p<.05, *η* ^2^ = 0.03). Two planned contrasts showed that the reaction times were significantly slower for incongruent than congruent primes when the response sound was present (sequences 1 to 6, the difference is 20.9 ms, F(1,32) = 24.86, p<0.0001), but were not significantly different between incongruent and congruent primes when the response sound is removed (sequences 7 to 12, the difference is 2.4 ms, F(1,32) = 0.00032, p = .98). Congruency did not interact with the groups (F(1,32) = 0.20, p = .66), nor with the response gestures (F(1,32) = 0.75, p = .39). Group One was significantly slower than Group Two (16.7 ms, F(1,32) = 7.0, p<.05, *η* ^2^ = 0.02), and there was a significant interaction between the groups and the sequences (F(11,352) = 2.48, p<.05, *η* ^2^ = 0.03). A closer inspection of the data showed that RTs decreased across sequences for Group One whereas they did not change across sequences for Group Two. This effect is orthogonal to our main interest and will not be further discussed. There was no main effect of the response gesture factor (F(1,32) = 0.04, p = .84), nor did it interact with any other factor. No three- or four-way interaction was significant.

Fig 7 also illustrates a number of observations that were tested with four post-hoc paired t-tests. First, two t-tests confirmed that whereas RTs for incongruent trials were slower than congruent trials in sequence 6 (last sequence with response sounds, 28.8 ms, t(32) = 4.01, p = <.01/4), there was no more priming effect as soon as response sounds were removed (sequence 7, first sequence with no response sounds, -3.2 ms, t(32) = -0.53, p = 0.601). Second, two other t-tests showed that: i) RTs were slower for all sequences with response sounds than for all sequences without response sounds for incongruent trials (the difference is 24.9 ms, t(32) = 5.44, p<.01/4); ii) they were not significantly different for congruent trials (the difference is 4.1 ms, t(32) = 0.91, p = .37). This shows that absence of a priming effect in the sequences with no response sounds was a result of decreases in RTs for incongruent trials.

### Accuracy

Overall, accuracy was high, with an average of 94.1%. Accuracy was submitted to the same ANOVA as previously described for RTs. Only congruency had a significant effect (F(2,64) = 26.7, p<.0001). Planned contrasts showed that accuracy was higher when there was no prime sound (96.0%) compared to incongruent primes (92.2%, F(1,32) = 60.9, p<.0001), and higher for congruent primes (94.1%) than incongruent primes (F(1,32) = 14.3, p<.001). There was no significant interaction between sequences and congruency, although there is a trend in the data for accuracy being higher for congruent than incongruent trials in sequences with a response sound (94.6% vs. 91.4%), and almost similar for both types of primes sequences without a response sound (93.6% vs. 93.0%).

### Discussion

The results of Experiment Five allow several conclusions. First, the response sounds are necessary to create the priming effect: the priming effect is not observable as soon as they are not systematically played for each key press (i.e. the gesture-sound association is not reinforced anymore). Second, these results also show that removing the response sounds has an asymmetrical effect on RTs for congruent and incongruent trials. Whereas RTs for congruent trials are not affected by removing the response sounds, RTs are decreased for congruent trials when the response sounds are removed. This strongly suggests that the priming effect was created by incongruent primes interfering with the response gestures, but not by congruent primes facilitating response gestures.

## General Discussion

As a whole, the experimental results confirmed the existence of an auditory motor priming phenomenon in which listening to a sound contingently associated with a gesture primes executing that gesture. Two findings define the conditions in which the priming effect did and did not occur. First, a similar priming effect was observed *both* for gesture-sound associations newly created by associative learning and for associations that were learned through life-long experience. Second, the priming effect was drastically reduced when the association was no longer being reinforced by the experiment. The following discussion examines these two findings and considers the influence of prior exposure to the gesture-sound associations and the plasticity of these associations.

The first clear finding is that long-term prior exposure to the gesture-sound association is *not necessary* to generate auditory-motor priming. Priming occurred both for recently learned arbitrary associations (key lifts triggering different sounds in Experiments One, Two and Five) and ecological gesture-sound associations learned through life-long exposure (commonly encountered gestures and the sounds they naturally produce in Experiment Three). Furthermore, the size of the priming effect was similar between Experiments Two (arbitrary) and Three (ecological), and even larger in Experiment One (key lifts with tones). If priming depended on the amount of prior experience with the gesture-sound associations we should have observed an effect at least somewhat smaller in Experiments One and Two (associations created within only a few-minutes) compared to Experiment Three (associations experienced during a lifespan).

The second notable finding is that long-term prior exposure to the gesture-sound association alone is *not sufficient* to generate auditory-motor priming to the full extent. Experiment Four used the same design as Experiment Three (tapping and scraping sounds and gestures) except that the response sounds produced by the gestures were muffled. Priming should not have been affected if it was mediated by a stable sound-gesture association established before the experiment. Instead, we found that the priming effect was greatly reduced (by about two thirds) in this version of the experiment. This shows that even if the gesture-sound association had been established prior to the experiment, it can be reconfigured rapidly (but not totally) as soon as the association is no longer reinforced. In addition, the observed priming effect could also have resulted from an association created *during* the experiment for *both* cases (arbitrary and ecological associations). Such an association would have been created by the training and learning phases, reinforced by each trial, and would disappear as soon as it is no longer reinforced. The results of Experiment Five confirm this interpretation: it is necessary to continuously reinforce the gesture-sound association for the prime sounds to influence the subsequent gesture. This shows that long-term prior exposure (e.g. processed through the ventral stream) is not sufficient to generate the priming effect.

But could it play some role in the phenomenon? It is important to consider that whereas the priming effect was eliminated in Experiment Five as soon as the association stopped being reinforced, it was only reduced in Experiment Four. Therefore, a part of the priming effect in Experiments Three and Four may have *also* resulted from life-long gesture-sound associations that could not be as quickly reconfigured.

Overall, these results demonstrate that the gesture-sound associations mediating auditory-motor priming are extremely plastic: they can be formed very quickly in the context of the experiment and reconfigured as quickly when the contingent association is violated, even if they had been established during life-long experience (we found little evidence for any special status for ecological associations). In other terms, a parsimonious interpretation of the auditory-priming phenomenon does not need long-term representations: the systematic playback of a sound after each gesture creates short-term associations that are able to interact with gesture selection, but almost disappear as soon as they are no longer reinforced (or when new associations take their place). Altogether, these results are strong indications of a type of memory representation that is flexible, in which older items are quickly replaced by new ones, akin to the traditional concept of short-term working memory and consistent with the properties of the dorsal stream of sensory processing.

The plasticity of the gesture-sound associations is consistent with other results reported in the literature. In addition to the response-effect compatibility mentioned previously, Brunel et al. (2013) showed that simply associating geometrical shapes and bursts of noise during a training phase was sufficient to prime the subsequent processing of tones, and that priming could transfer to different members of the categories of geometrical shapes that had not been previously associated with sounds [57]. In a related study, Zmigrod and Hommel (2009) showed that responding to a tone with a key press could create an association that was strong enough to slow down participants’ reaction when they had to immediately perform the same response to a different tone (and/or a different action in response to the same tone) [58]. Although this phenomenon was not the priming of an action generated by a sound, the flexibility of this association is consistent with our findings.

The result that gesture-sound associations can be quickly relearned is also in line with some results found for visuo-motor priming [50]. Incompatible training created unrealistic visuo-motor associations that replaced or weakened preexisting associations reinforced during the initial phase. As in our results, the representations that mediate priming can be easily changed by simple exposure to new associations. This is also consistent with the experiment reported by Bourquin et al. (2013) [59]. They showed that motor evoked potentials of hand muscles increased when participants first listened to sounds of manual actions, but decreased with repeated exposure to the sounds (i.e. repetition suppression). Note however that Elsner and Hommel (2001) found that participants freely pressing a key in response to a tone responded more quickly when they selected the key that had been previously associated with the tone, even if the key was no longer producing that tone, suggesting that the gesture-sound association is resistant to extinction of the reinforcement [60]. However, the difference between paradigms complicates a direct comparison.

The minor role of prior exposure in modulating the priming effect is also compatible with the theory of event coding (TEC). The TEC suggests an interpretation whereby the task relevance of features (of both the stimuli and of the response) are the major determinants of the priming effects. TEC’s common representational domain for stimuli and goals would predict that the sound resulting from an action (a perceived event) activates a common representation that is also responsible for planning that action (intended event) [45]. The flexible representation could produce this effect for both newly-learned and well-learned associations and it would not necessarily differentiate between artificial and ecologically-plausible stimuli. The theory of event coding posits that the different dimensions of a gesture-sound association can be weighted differently according to their task relevance and the participant’s intentions (intentional weighting [46]). When the actions stop producing sounds as in Experiments Four and Five the acoustic dimension of the association becomes completely task-irrelevant and thus loses its ability to prime the action. However, TEC also postulates that the process of intentional weighting is slow and “sticky” (takes time to be reconfigured). Whereas the TEC is consistent with the priming effect we observed, whether or not it is also consistent with the the rapid loss of priming will depend on the degree of “stickiness” that is assumed for its flexible feature/dimension weighting. Testing such an idea would however require a different design that manipulates for instance the task relevance of the prime.

These interpretations still do require some sort of short-term memorization. Auditory codes (and other features of the gestures) need to be at least temporarily activated in a buffer and associated with the motor plans of the two possible gestures (e.g. an “auditory event file” [45, 58, 61]). Such a temporary buffer would need to store representations of different natures (sensory codes from different modalities and motor plans). Such a buffer could also activate sensory-motor representations retrieved from long-term memory, such as when presenting the name of an object is sufficient to prime an action [22].

The idea of a temporary buffer receiving and coordinating representations from different sensory modalities, motor plans, and semantic information is consistent with some views of the function of the left parietal lobe. For instance, [62] proposed that regions of the parietal lobe acts as a hub connected to the ventral premotor cortex and posterior inferior frontal gyrus (IFG) via dorsal fibers along the superior longitudinal fascicle and to the anterior part of the IFG via ventral fibers. They suggested the angular gyrus may act as an interface between ventral and dorsal streams that coordinates cognitive control, perception, and action. (Note that studies of patients suffering from aphasia and apraxia have also shown that cortical regions at the junction of the left temporal and parietal lobes play an fundamental role in the semantic processing of non-verbal sounds, and especially actions sounds [63, 64].) Rauschecker and Scott have also proposed a similar idea [16].

Such interpretations are speculative and further work is needed to characterize the auditory priming effect. As with the study of visually guided actions, the question of time and memory is crucial. Further work will reveal how the effect unfolds over time. For instance, introducing a time delay between the learning and test phases (see for instance [65]) or using using incompatible training as in [50] will further characterize the auditory-motor priming effect. An important distinction between the effects of our priming sounds on actions and visually guided actions is that preceding sounds do not normally guide actions. Despite the fact that environmental sounds usually happen as a consequence of an action, reversing the temporal order of sound and actions nonetheless produces priming. This clearly highlights the *bidirectional* nature of the tight link between sounds and gestures.

## Conclusion

In summary, the results of these experiments have confirmed that playing a sound associated with an action is capable of priming the planning of that action via recent association. This effect can be reproduced as readily with ecological associations learned through life-time exposure (e.g., a tapping action produces an impact sound) as it can with newly-learned arbitrary associations (e.g., lifting a key produces a tone). Moreover, priming nearly disappears when the association ceases to be reinforced. Auditory-motor priming provides evidence that even long-term relationships between sounds and gestures are extremely plastic, and can be readily established and readily reconfigured.
